# Semiautomatic Segmentation of Ventilated Airspaces in Healthy and Asthmatic Subjects Using Hyperpolarized ^3^He MRI

**DOI:** 10.1155/2013/624683

**Published:** 2013-03-31

**Authors:** J. K. Lui, A. S. LaPrad, H. Parameswaran, Y. Sun, M. S. Albert, K. R. Lutchen

**Affiliations:** ^1^Boston University, School of Medicine, Boston, MA 02118, USA; ^2^Department of Biomedical Engineering, College of Engineering, Boston University, Boston, MA 02115, USA; ^3^Department of Radiology, Brigham and Women's Hospital, Boston, MA 02115, USA; ^4^Department of Radiology, University of Massachusetts Medical School, Worcester, MA 01655, USA; ^5^Dana Farber Cancer Institute, Boston, MA 02115, USA; ^6^Department of Chemistry, Lakehead University, Thunder Bay, ON, Canada P7A 5E1; ^7^Thunder Bay Regional Research Institute, Thunder Bay, ON, Canada P7B 6V4

## Abstract

A segmentation algorithm to isolate areas of ventilation from hyperpolarized helium-3 magnetic resonance imaging (HP ^3^He MRI) is described. The algorithm was tested with HP ^3^He MRI data from four healthy and six asthmatic subjects. Ventilated lung volume (VLV) measured using our semiautomated technique was compared to that obtained from manual outlining of ventilated lung regions and to standard spirometric measurements. VLVs from both approaches were highly correlated (*R* = 0.99; *P* < 0.0001) with a mean difference of 3.8 mL and 95% agreement indices of −30.8 mL and 38.4 mL. There was no significant difference between the VLVs obtained through the semiautomatic approach and the manual approach. A Dice coefficient which quantified the intersection of the two datasets was calculated and ranged from 0.95 to 0.97 with a mean of 0.96 ± 0.01 (mean ± SD). VLVs obtained through the semiautomatic algorithm were also highly correlated with measurements of forced expiratory volume in one second (FEV_1_) (*R* = 0.82; *P* = 0.0035) and forced vital capacity (FVC) (*R* = 0.95; *P* < 0.0001). The technique may open new pathways toward advancing more quantitative characterization of ventilation for routine clinical assessment for asthma severity as well as a number of other respiratory diseases.

## 1. Introduction

Recent advancements in hyperpolarized helium-3 magnetic resonance imaging (HP ^3^He MRI) enable direct visualization of ventilation in the lung [1, 2]. While normally ventilated lungs have been found to exhibit a homogeneous distribution of gas signal, obstructed lungs such as in asthma show areas of signal depletion, often referred to as ventilation defects [1–5]. It is increasingly accepted that quantifying spatial patterns in the ventilation distribution can provide rich insight on the severity of asthma and how well a specific patient responds to a prescribed therapy [1, 2]. Additionally, such information may provide novel perspectives in the fundamental nature of asthma with regard to whether it is a localized airway pathology or a global lung disease.

Traditional analysis of HP ^3^He MRI has primarily been qualitative in nature, largely restricted to a scoring system that required a radiologist to visually estimate the number of ventilation defects [1–4]. These approaches were subjective and were likely inconsistent and time intensive. A number of quantitative methods have emerged for the segmentation of ventilated airspaces. Initial attempts by Kauczor et al. [6] relied on a thresholding scheme which assumed a Gaussian distribution of noise. However, such an assumption leads to an approximately 60% underestimation of the true noise power [7]. Later efforts by Tzeng et al. [5] and Woodhouse et al. [8] applied a threshold value that relied on a signal-to-noise threshold but still required rigorous manually outlined lung boundaries. More recent work using class-based algorithms with lung partitioning using a Gaussian mixture model [9] and methods that employ fuzzy C-means and K-means clustering [10–12] have also been introduced. These methods were automated but required additional manual removal of the trachea. By default, the trachea and associated large airways comprise a majority of the anatomic dead space which contains the largest percentage of HP ^3^He gas [5]. Since our goal was to target gas exchange regions, removal of the trachea and associated large airways would result in a more accurate assessment of ventilation.

In this study, we introduced a robust, semiautomatic algorithm for rapid segmentation of HP ^3^He MRI into distinct regions based on ventilation. The ventilated lung volume (VLV) quantified using our method was compared to that measured using a conventional manual analysis by a trained technician to determine the accuracy of our segmentation. As spirometry still remains as the gold standard for measurement of airway obstruction, we compared measurements of lung volume from HP ^3^He MRI using our method to forced expiratory volume in one second (FEV_1_) and forced vital capacity (FVC). The scope of this paper is to introduce the methodology and a preliminary study with data from four healthy and six asthmatic subjects. The intent is to provide proof-of-principle, in a fashion that indicates the capability of this approach in analyzing spatial distributions for ventilation [5, 12, 13] and future modeling studies [14, 15] for asthma [1–5] as well as the potential to be streamlined to other respiratory diseases such as chronic obstructive pulmonary disease [8, 11, 12] and cystic fibrosis [11, 16, 17].

## 2. Materials and Methods

### 2.1. Subject Enrollment

The Health Insurance Portability and Accountability Act-Compliant research protocol in this study was approved by both Boston University and Brigham and Women's Hospital Institutional Review Boards. Written informed consent was obtained from all recruits, which consisted of four healthy subjects (two men and two women: age range 21–23 years; mean age 22 years) and six asthmatic subjects (one man and five women: age range 19–23 years; mean age 22 years). Before the first study visit, each subject participated in a screening day visit during which a methacholine challenge was administered to determine a PC_20_ dose that elicited a 20% drop in baseline FEV_1_. This index was used to separate healthy from asthmatic subjects. For our protocol, healthy subjects were nonsmokers with no history of respiratory diseases and exhibited PC_20_ values of >25 mg/mL. Asthmatic subjects consisted of those with a history of asthma who exhibited PC_20_ values of <8 mg/mL. The demographics are detailed in Table 1.

### 2.2. Image Acquisition Protocol

Standard spirometry measurements were recorded with the subject in supine position. Each subject was instructed to inhale a ~1 liter mixture of ~33% HP ^3^He-67% N_2_ from functional residual capacity (FRC). Images were acquired on a General Electric Signa LX 1.5 MRI scanner equipped with a heterodyne system which included frequency mixers to image at the ^3^He NMR frequency of 48.65 Hz. The system interfaced with a flexible quadrature lung coil (Clinical MR Solutions, Brookfield, WI) tuned to the same frequency. Hyperpolarization of the ^3^He gas was initiated through a collision spin exchange with vaporized rubidium optically pumped using a custom-built polarizer. The scans employed a Fast Gradient Echo pulse sequence that compiled coronal multislice images with a field of view (FOV) of 46 cm, 128 × 256 matrix dimensions (zero-padded to 256 × 256), 13 mm slice thickness, 0 mm gap between slices, 1.8 mm in-slice resolution, 31.25 kHz bandwidth, 14–18° flip angle, TE/TR 1.228 ms/50–75 ms, and interleaved data acquisition. Typically, 8–14 slices were obtained for each subject, depending on the anterior to posterior depth of the lung.

### 2.3. MR Image Processing

**Figure 1:**
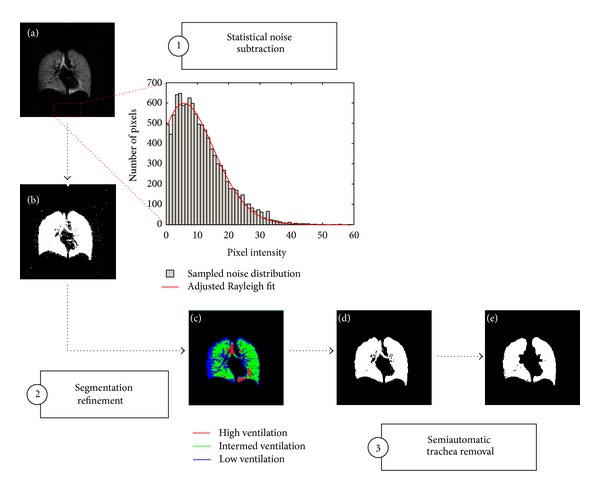
Detailed schematic of semiautomatic segmentation algorithm. The example shown here is from a healthy subject. The first step is a statistical noise subtraction to generate an initial binary mask of the input image (a). Thereafter, the resultant lung mask (b) is refined through a four-class FCM clustering which partitions the entire image into four categories: negligible ventilation, low ventilation, intermediate ventilation, and high ventilation (c). Pixels that fall within the negligible ventilation class are subsequently discarded to form a corrected mask (d). Through a semiautomatic trachea removal involving a seeded region-growing algorithm, an area filter for connectivity, and a series of morphological operations, a final binary image representative of ventilated airspaces is obtained (e).

A detailed schematic of our semiautomatic segmentation method is illustrated in Figure 1. Our methods will refer to various panels in Figure 1. There are three steps to our semiautomatic segmentation method. (1) A preprocessing routine is applied involving statistical noise subtraction. (2) The image pixels are correspondingly clustered into ventilation classes to refine our initial segmentation. (3) The trachea and major airways are removed to obtain a final binary image representative of ventilated airspaces.

#### 2.3.1. Statistical Noise Subtraction

HP ^3^He MR images were first preprocessed through a denoising scheme by determining an optimal threshold from a sampled background noise distribution located outside of the lung field. This space comprised an automated 25 × 50 pixel box in the bottom center of each image slice (Figures 1(a) and 1(b)). The distribution is fitted through a nonlinear regression with an adjusted Rayleigh curve

(1) $$\begin{array}{c} r\left(f\right)=\left(\alpha f+\delta \right)\frac{e^{-{\left(\alpha f+\delta \right)}^{2}/2\sigma^{2}}}{\sigma^{2}}, \end{array}$$

where *f* is the intensity of background noise, with parameters *σ* and *α*. In contrast to a similar technique previously applied in brain tissue segmentation [18, 19], our approach employed an additional shifting parameter, *δ*, which accounted for horizontal shifts in curve-fitting and provided a much stronger fit to the sampled data. An optimal threshold, *τ*_*n*_, was subsequently derived from the minimization of an error term

(2) $$\begin{array}{c} \varepsilon_{\tau }=\sum_{f=0}^{\tau_{n}-1}g\left(f\right)+\sum_{f=\tau_{n}}^{\infty }r\left(f\right), \end{array}$$

where the function, *g*(*f*), constituted the subtracted distribution calculated by the difference between the best-fit adjusted Rayleigh curve, *r*(*f*), and the pixel intensity distribution of the sampled background noise, *h*(*f*). Consider

(3) $$\begin{array}{c} g\left(f\right)=r\left(f\right)-h\left(f\right). \end{array}$$

The purpose of the preprocessing was to automatically remove discernible sites of noise artifacts to construct an initial binary mask (see Figure 1(a)).

#### 2.3.2. Segmentation Refinement

Pixel intensities across the entire image space were correspondingly partitioned through a clustering scheme. Here, we describe the clustering using fuzzy C-means (FCM) clustering [10, 11]. However, this step can also be replaced by a K-means clustering algorithm [12, 13] as both these algorithms use the same cost function. Briefly, the algorithm initializes four random cluster centers in which a corresponding membership function, *u*_*ik*_, is calculated. The membership function is based on a distance measure which describes the degree of similarity between each data point and each cluster center given by

(4) $$\begin{array}{c} u_{ik}=\frac{1}{\sum_{j=1}^{C}{\left(D_{ik}/D_{jk}\right)}^{2/\left(m-1\right)}}, \end{array}$$

where *C* is the number of distinct clusters, and *m* ∈ [0, *∞*) is a weighing parameter used to control the level of fuzziness in the classification scheme, typically initialized to 2 [20]. The variables, *D*_*ik*_ and *D*_*jk*_, constitute the distance between point *k* to the cluster center of clusters *i* and *j*, respectively. From the resultant calculation of the membership, *u*_*ik*_, a new cluster center for each class, *c*_*j*_, is calculated across all data points, given by the following relationship [20]:

(5) $$\begin{array}{c} c_{j}=\frac{\sum_{i=1}^{N}u_{ik}^{m}\cdot x_{i}}{\sum_{i=1}^{N}u_{ik}^{m}}. \end{array}$$

Using these new cluster centers, *c*_*j*_, the membership *u*_*ik*_ is updated, and the process is iteratively repeated, based on minimization of the following objective function [20]:

(6) $$\begin{array}{c} J_{m}=\sum_{i=1}^{C}\sum_{k=1}^{N}{\left(u_{ik}\right)}^{m}D_{ik}. \end{array}$$

A predefined criterion, *ε*, between 0 and 1, is set such that when reached, the algorithm is terminated. Following previous publications [10, 11], we split the ventilated lung region into four clusters that corresponded to negligible ventilation, low ventilation, intermediate ventilation, and high ventilation (Figures 1(c) and 1(d)). Since our eventual areas of interest comprised ventilated regions within the lung, pixel intensities designated to the negligible ventilation class were treated as part of the background.

#### 2.3.3. Semiautomatic Trachea Removal

Typically, we acquired 8–14 image slices anterior to posterior for each subject. Some of these images contain the trachea and the main stem bronchi, particularly in the middle slices, which, by default, hold the largest percentage of HP ^3^He gas. Since the trachea and associated large airways are not directly involved in gas exchange, it became crucial to remove them for an accurate assessment of ventilation (Figure 1(e)). Therefore, we employed a slice-by-slice seeded region-growing algorithm [21, 22] which detected edges based on the intensity levels of connected pixels.

In its simplest form, the technique requires an initiation point, known as a seed, which is often manually selected by the user [21, 22]. Each connected component of the seed is then flagged, and a difference measure is calculated by a predefined criterion at each iteration. The goal of the algorithm is to enable a final segmentation of regions as homogeneous as possible while constrained by each pixel's connectivity to the initial seed point. A basic model was defined by Adams and Bischof, using a running mean calculated at each iteration starting at a designated seed point [21]. Given *T* as the set of all as-yet unallocated pixels which border at least one of the regions,

(7) $$\begin{array}{c} T=\left\{x\notin {⋃}_{i=1}^{n}A_{i}\;|\;N\left(x\right)\cap {⋃}_{i=1}^{n}A_{i}\neq 0\right\}. \end{array}$$

The difference measure, *δ*(*x*), bound by the running mean is described by the following expression, where *g*(*x*) is the gray value of the image point *x*. Consider

(8) $$\begin{array}{c} \delta \left(x\right)=\left|g\left(x\right)-\underset{y\in A_{i(x)}}{\text{mean}}\left[g\left(y\right)\right]\right|. \end{array}$$

From the set of unallocated pixels, *T*, which border at least one of the regions connected to the seed point, a minimum distance, *δ*(*z*), was set as the segmented space [21]. Consider

(9) $$\begin{array}{c} \delta \left(z\right)=\underset{x\in T}{\min}\left\{\delta \left(x\right)\right\}. \end{array}$$

**Figure 2:**
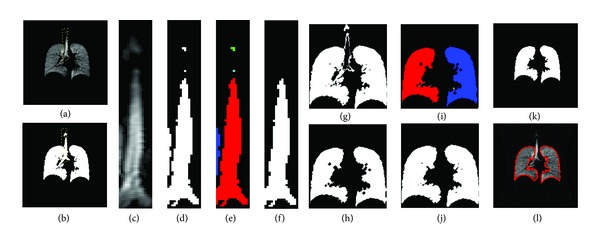
Semiautomatic trachea removal. The example shown here is from an asthmatic subject. A corresponding HP ^3^He MRI image (a) and a binary ventilation image (b) are displayed in which a user-selected bounding box captures the trachea (c). An initial seed point is selected within the trachea, and a region-growing algorithm is applied to yield a resultant binary extraction (d). Thereafter, each element within the image space is labeled based on connectivity (e), and an area filter is used to isolate the trachea (f). A simple binary subtraction between the isolated trachea is then used (g) followed by a binary erosion (h). Then, each element within the image space is labeled again based on connectivity to isolate the right and the left lungs (i), and a binary dilation is thereafter applied (j)-(k) with a corresponding HP ^3^He MRI showing the outlined boundaries (l).

A detailed schematic is illustrated in Figure 2. The technique was knowledge based and required two inputs: a user-defined bounding box to limit the processing space to the trachea and an initial seed point composed of a single pixel manually selected inside the trachea (Figures 2(a)–2(c)). Following interrogation of each pixel within the isolated bounding box, an outlined space was obtained (Figure 2(d)). Each element within the image space was correspondingly labeled based on connectivity (Figure 2(e)), and an area filter was applied to isolate the trachea (Figure 2(f)). The area filter was based on pixel connectivity in which connected areas of fewer than 50 pixels were selectively removed. A simple binary subtraction between the input (with the trachea) and output images (without the trachea) yielded a binary image with the trachea selectively removed (Figure 2(g)).

We discarded residual artifacts from the crude binary image subtraction through morphological operations. A binary erosion (Figure 2(h)) was first used. Then, each connected element was labeled, and another area filter was applied to selectively remove connected areas of fewer than 50 pixels (Figure 2(i)). Finally, a binary dilation was applied (Figure 2(j)). The rationale was to target weakly connected areas usually comprising the larger associated airways extending from the main stem bronchi. The details on the operators are outlined by Serra [23]. Briefly, given the mask as a discrete Euclidian image, *A*(*m*, *n*) ∈ *Z* ^2^, dilation of *A* by a structural element, *B*, is expressed as follows:

(10) $$\begin{array}{c} A⊕B=\left\{c\;|\;c=a+b,a\in A,b\in B\right\}. \end{array}$$

The erosion of *A* by *B* is given as

(11) $$\begin{array}{c} A⊖B=\left\{c\;|\;{\left(B\right)}_{c}\subseteq A\right\}. \end{array}$$

**Figure 3:**
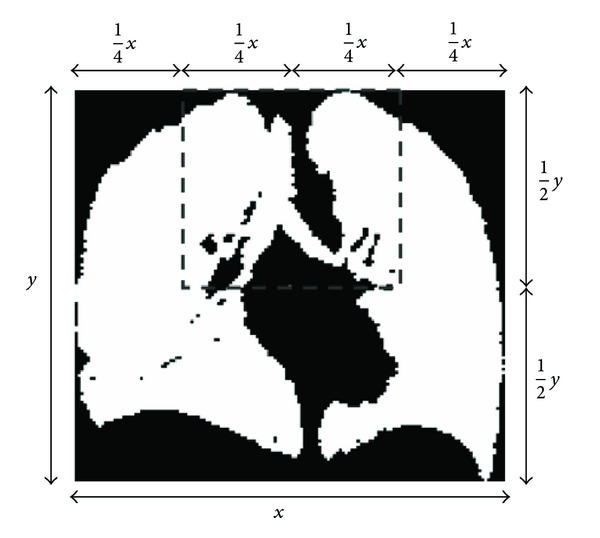
Template for morphological operations. Bounding box shown in gray illustrates the boundaries to which morphological operations were applied based on one-quarter of the width (*x*) and one-half the height (*y*) from the centroid.

For our processing scheme, we used a disk structural element for both binary erosion and binary dilation. We applied these operations to a fixed template based on the maximum width and height of the segmented lung slice as illustrated in Figure 3. This was done to maximize removal of the larger attached airways while minimizing morphological distortion particularly along the concave lung base.

### 2.4. Statistical Data Analysis

To assess the accuracy of our method with the manual analysis currently employed to assess ventilation heterogeneity from HP ^3^He MRI, all images were processed by a trained lab technician (5 years experience with HP ^3^He MRI) using a MATLAB-coded software (MathWorks, Natick, MA). In the manual analysis, lung contours and ventilation defects were outlined manually, and the VLV was calculated from the number of the pixels in the regions identified as being ventilated. A paired *t*-test was used to compare the VLVs from the manual and semiautomated methods. An unpaired *t*-test was used to compare the VLVs between the healthy and the asthmatic subjects. A Dice coefficient was also calculated to measure the agreement or similarity between the VLV using our approach, *A*, and the manual approach, *B* [24]. The Dice coefficient ranges from 0 to 1, with 1 indicating perfect agreement. Consider

(12) $$\begin{array}{c} \text{Dice}\left(A,B\right)=\frac{2\left|A\cap B\right|}{\left|A\right|+\left|B\right|}. \end{array}$$

We performed a linear regression analysis across all subjects and calculated a correlation coefficient and the slope between semiautomatic and manual methods. Bland Altman analysis [25] was used to determine the 95% limits of agreement calculated from the mean and standard deviation of the volume difference between the two methods of segmentation. VLVs through our algorithm were compared to PFTs, specifically, to FEV_1_ and FVC, functional measures that vary with the size and level of lung obstruction. Finally, an unpaired *t*-test was also used to compare FEV_1_ and FVC between the healthy and asthmatic subjects. 

## 3. Results

**Figure 4:**
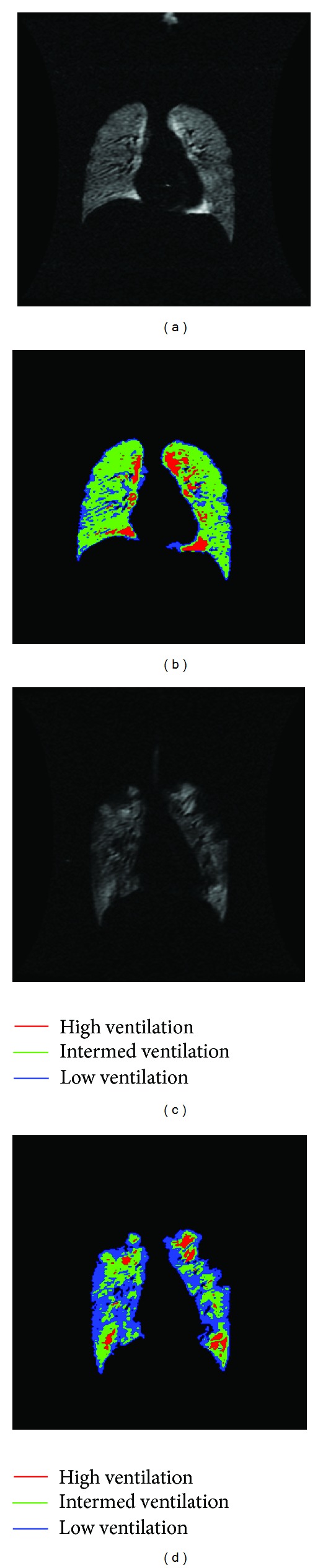
Segmented lung volumes for a healthy and an asthmatic subject. Panels (a) and (c) show a slice of the HP ^3^He MRI for a healthy and an asthmatic subject, respectively. The corresponding segmented images are divided into clusters of high, intermediate, and low ventilation as shown in panels (b) and (d). Note the increased predominance in pockets of hypointense areas indicating low ventilation in the asthmatic subject as compared to the healthy subject.

A typical segmentation of the ventilated regions in the HP ^3^He MRI into three distinct classes of ventilation is shown in Figure 4. In our limited subset of six asthmatics and four healthy subjects, the asthmatics showed increased predominance in pockets of hypointense areas indicating low ventilation in the asthmatics as compared to the healthy subjects. We compiled a total of 109 coronal slices for ten subjects. For healthy subjects, segmentation with the semiautomatic approach yielded a mean VLV of 3.88 ± 0.75 L (mean ± SD), compared to the manual approach, which gave a mean VLV of 3.90 ± 0.72 L. For asthmatics, the mean VLVs of the semiautomatic approach were 3.83 ± 1.11 L and 3.85 ± 1.17 L with the manual approach. There was not a statistically significant difference in FEV_1_ between the semiautomatic and manual approaches (*P* = 0.41). The resulting dice coefficients for each subject are illustrated in Table 2. The coefficients ranged from 0.95 to 0.97 with a mean of 0.96 ± 0.01.

**Figure 5:**
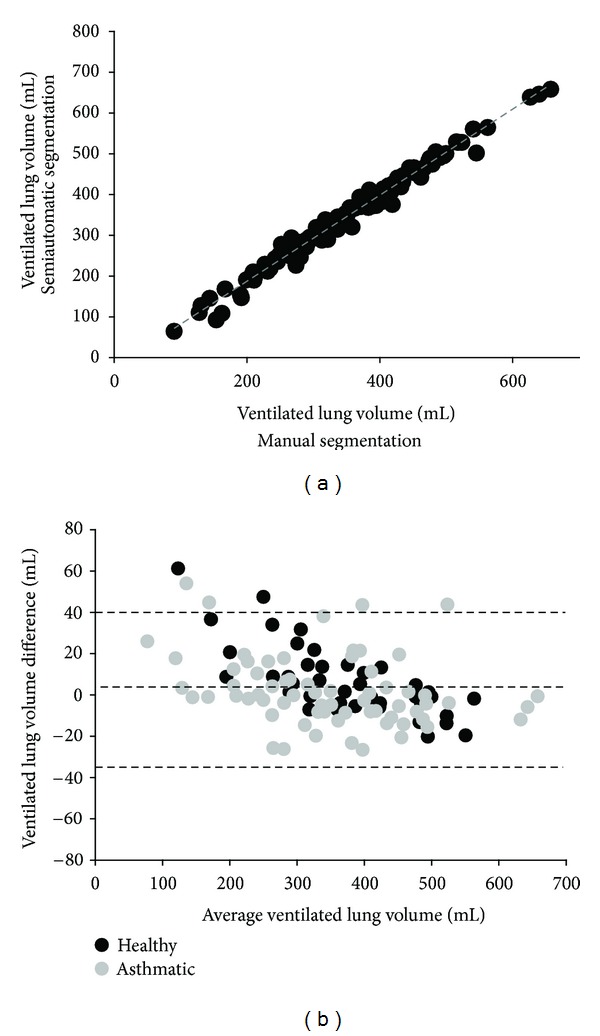
Comparison of VLV measured using semiautomatic and manual segmentation. (a). A high correlation was observed between the VLVs obtained between both manual and automated methods (*R* = 0.99, *P* < 0.0001) (b). A Bland-Altman analysis resulted in a mean VLV difference of 3.8 ± 17.3 mL with lower and upper 95% limits of agreement of −30.8 mL and 38.4 mL, respectively.

**Figure 6:**
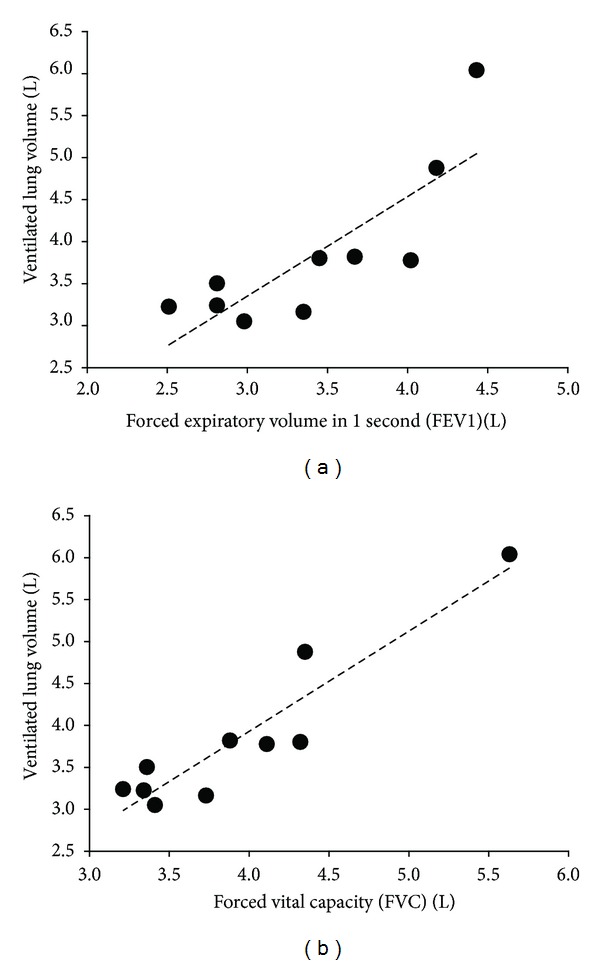
Scatter plot of ventilated lung volumes versus measurements of FEV_1_ (a) and FVC (b). Comparison to both measurements yield positive trend lines with strong association to FVC (*R* = 0.93, *P* = 0.0035) and FEV_1_ (*R* = 0.84, *P* < 0.0001).

Across each coronal slice of the lung, VLV measurements obtained through both methods were highly correlated (*R* = 0.99; slope = 1.1; *P* < 0.0001) (Figure 5(a)). From the Bland-Altman analysis, the mean VLV difference was 3.8 ± 17.3 mL. The lower and upper 95% limits of agreement were −30.8 mL and 38.4 mL, respectively (Figure 5(b)). Comparison to spirometry yielded a high correlation to measurements of FVC (*R* = 0.93; slope = 1.21; *P* < 0.0001) (Figure 6(a)) and FEV_1_ (*R* = 0.84; slope = 1.22; *P* = 0.0035) (Figure 6(b)). For healthy subjects, mean FEV_1_ was 3.71 ± 0.53 L and mean FVC was 3.94 ± 0.40 L; for the asthmatic subjects, mean FEV_1_ was 3.23 ± 0.69 L and mean FVC was 3.93 ± 0.92 L. There was no statistically significant difference in FEV_1_ (*P* = 0.27) and FVC (*P* = 0.99) between the healthy and asthmatic subjects.

## 4. Discussion

To this day, HP ^3^He MRI has confirmed and advanced a number of new perspectives in asthma. For one, when exposed to airway smooth muscle provocation, the lungs will constrict heterogeneously with the number and size of ventilation defects directly correlating to the level of clinical severity [1–4]. In cases of very severe asthma, heterogeneously distributed ventilation defects may be even present at baseline [1, 2]. More recently, there is even some evidence that the size and location of many of these ventilation defects in asthmatic lungs tend to not change with time or repeated bronchoconstriction [1, 3]. These notions are primarily qualitative as they are based on visual inspection of ventilation images. However, together they raise intriguing clinical and structure-function questions regarding whether one could apply a quantitatively robust method for diagnosing the severity of baseline asthma and for evaluating the efficacy of treatment.

Up until now, much effort has been devoted to extracting detailed structural information from HP ^3^He MRI. While qualitative methods of analysis [1–4] have raised the concerns about consistency, quantitative methods [5, 6, 8–13] have paved new insights in the characterization of ventilation. However, many of these segmentation approaches did not include an extraction and removal of the trachea and mainstem bronchi with associated large airways [5, 6, 8, 10–13]. To our knowledge, our approach is the first to enable both a segmentation of ventilated airspaces and a direct selective removal of these components that constitute the anatomic dead space. When we applied our method to just a small pilot-study number of healthy and asthmatic subjects, we did not find a statistically significant difference in the VLV between our semiautomatic method and our manual tracings that served as the ground truth for our analytical comparisons. The results obtained from our method were able to produce high correlations to those obtained through manual processing and showed high degree of similarity and agreement through the Dice coefficients and Bland Altman analysis, respectively. 

However, there are some limitations to our technique. Static scans do not represent real-time ventilation but instead represent snapshots in real time. True ventilation would require a multibreath technique [26] necessitating a greater amount of ^3^He gas. Hence, our technique was only capable of calculating a total VLV based on the gas distributive patterns at breath-hold. Another limitation is the dependence in user input in the semiautomatic trachea removal. Varying the size of the bounding box can certainly impact the semiautomatic trachea removal algorithm. A crucial element of the approach is application of an area filter that isolates the trachea. If a large region outside the trachea was chosen, then it would indeed be more difficult to adjust the area filter to discard small regions of connected pixels and large regions of connected pixels as opposed to the status quo of just removal of small regions. The seed point, we believe, should not impact the trachea because, for the most part, it is nearly homogenous in signal intensity. However, sensitivity studies in varying both the seed point and the bounding box can certainly be done in the future. A final limitation was in the thickness of the slices. Because these images were acquired at breath-hold, thick slices of 13.13 mm were compiled in order to cover the entire extent of the lung while trying to minimize discomfort suffered by the subject. To compare lung volumes between each subject, we recommend the use of ^1^H proton MRI scans to determine the volume of the thoracic cavity to normalize for lung size.

## 5. Conclusion

In conclusion, our work outlines a novel statistically and quantitatively driven imaging analysis that may provide a powerful and valuable additional tool for the clinical assessment of asthma severity. With the emergence of modeling approaches to combine imaging modalities to construct patient-specific models [14, 15], segmentation of lung ventilation becomes more important than ever. These methods may provide new perspectives in structure-function relations and hold the potential to be extrapolated to other respiratory diseases. 

## Figures and Tables

**Table 1:** Subject demographics and spirometry measurements.

Subject	Sex, M/F	Age, yr	Height, cm	Weight, kg	BMI	FEV_1_, L	FEV %Pred	FVC, L	FEV_1_/FVC	FEV_1_/FVC %Pred	PC_20_
Healthy											
H1	F	23	153	44	18.8	2.98	100	3.41	87.4	101	>25
H2	F	22	175	68	22.2	3.67	109	3.88	94.6	108	>25
H3	M	23	180	82	25.2	4.18	88	4.35	96.1	115	>25
H4	M	21	189	73	20.4	4.02	79	4.11	97.8	116	>25

Asthmatic											
A1	F	19	155	54	22.3	2.81	87	3.36	83.6	93	0.12
A2	F	23	157	61	24.7	2.81	91	3.21	87.5	101	0.17
A3	F	21	163	73	27.5	3.35	102	3.73	89.8	104	1.11
A4	M	23	188	93	26.4	4.43	86	5.63	78.7	94	0.17
A5	F	22	172	80	26.9	3.45	94	4.32	79.9	92	3.31
A6	F	22	157	55	22.1	2.51	80	3.34	75.1	87	0.12

**Table 2:** Summary of ventilated lung volumes measurements and the corresponding dice coefficients.

	VLV Semiautomatic segmentation (L)	VLV Manual segmentation (L)	Dice coefficient
Healthy			
H1	3.05	3.14	0.96
H2	3.82	3.73	0.97
H3	4.88	4.87	0.96
H4	3.78	3.84	0.96

Asthmatic			
A1	3.50	3.55	0.96
A2	3.24	3.19	0.97
A3	3.16	3.14	0.96
A4	6.04	6.19	0.96
A5	3.80	3.80	0.96
A6	3.23	3.23	0.95

## References

[1] de Lange EE, Altes TA, Patrie JT (2009). Changes in regional airflow obstruction over time in the lungs of patients with asthma: evaluation with ^3^He MR Imaging. *Radiology*.

[2] De Lange EE, Altes TA, Patrie JT (2006). Evaluation of asthma with hyperpolarized helium-3 MRI: correlation with clinical severity and spirometry. *Chest*.

[3] de Lange EE, Altes TA, Patrie JT (2007). The variability of regional airflow obstruction within the lungs of patients with asthma: assessment with hyperpolarized helium-3 magnetic resonance imaging. *Journal of Allergy and Clinical Immunology*.

[4] Samee S, Altes T, Powers P (2003). Imaging the lungs in asthmatic patients by using hyperpolarized helium-3 magnetic resonance: assessment of response to methacholine and exercise challenge. *Journal of Allergy and Clinical Immunology*.

[5] Tzeng Y-S, Lutchen K, Albert M (2009). The difference in ventilation heterogeneity between asthmatic and healthy subjects quantified using hyperpolarized ^3^He MRI. *Journal of Applied Physiology*.

[6] Kauczor HU, Markstaller K, Puderbach M (2001). Volumetry of ventilated airspaces by ^3^He MRI: preliminary results. *Investigative Radiology*.

[7] Gudbjartsson H, Patz S (1995). The rician distribution of noisy MRI data. *Magnetic Resonance in Medicine*.

[8] Woodhouse N, Wild JM, Paley MNJ (2005). Combined helium-3/proton magnetic resonance imaging measurement of ventilated lung volumes in smokers compared to never-smokers. *Journal of Magnetic Resonance Imaging*.

[9] Tustison NJ, Avants BB, Flors L (2011). Ventilation-based segmentation of the lungs using hyperpolarized ^3^He MRI. *Journal of Magnetic Resonance Imaging*.

[10] Cooley B, Acton C, Salerno M Automated scoring of hyperpolarized helium-3 MR lung ventilation images: initial development and validation.

[11] Ray N, Acton ST, Altes T, De Lange EE, Brookeman JR (2003). Merging parametric active contours within homogeneous image regions for MRI-based lung segmentation. *IEEE Transactions on Medical Imaging*.

[12] Kirby M, Heydarian M, Svenningsen S (2012). Hyperpolarized ^3^He magnetic resonance functional imaging semiautomated segmentation. *Academic Radiology*.

[13] Kirby M, Matthew L, Heydarian M, Etemad-Rezai R, McCormack DG, Parraga G (2011). Chronic obstructive pulmonary disease: quantification of bronchodilator effects by using hyperpolarized He MR imaging. *Radiology*.

[14] Campana L, Kenyon J, Zhalehdoust-Sani S (2009). Probing airway conditions governing ventilation defects in asthma via hyperpolarized MRI image functional modeling. *Journal of Applied Physiology*.

[15] Mullally W, Betke M, Albert M, Lutchen K (2009). Explaining clustered ventilation defects via a minimal number of airway closure locations. *Annals of Biomedical Engineering*.

[16] Donnelly LF, MacFall JR, McAdams HP (1999). Cystic fibrosis: combined hyperpolarized ^3^He-enhanced and conventional proton MR imaging in the lung—preliminary observations. *Radiology*.

[17] McMahon CJ, Dodd JD, Hill C (2006). Hyperpolarized ^3^Helium magnetic resonance ventilation imaging of the lung in cystic fibrosis: comparison with high resolution CT and spirometry. *European Radiology*.

[18] Atkins MS, Mackiewich BT (1998). Fully automatic segmentation of the brain in MRI. *IEEE Transactions on Medical Imaging*.

[19] Brummer ME, Mersereau RM, Eisner RL, Lewine RRJ (1993). Automatic detection of brain contours in MRI data sets. *IEEE Transactions on Medical Imaging*.

[20] Bezdek JC, Keller J, Krisnapuram R, Pal NR (1999). *Fuzzy Models and Algorithms for Pattern Recognition and Image Processing*.

[21] Adams R, Bischof L (1994). Seeded region growing. *IEEE Transactions on Pattern Analysis and Machine Intelligence*.

[22] Mancas M, Gosselin B, Macq B Segmentation using a region growing thresholding.

[23] Serra J (1982). *Image Analysis and Mathematical Morphology*.

[24] Dice LR (1945). Measures of the amount of ecologic association between species. *Ecology*.

[25] Bland JM, Altman DG (1986). Statistical methods for assessing agreement between two methods of clinical measurement. *The Lancet*.

[26] Deninger AJ, Månsson S, Petersson JS (2002). Quantitative measurement of regional lung ventilation using ^3^He MRI. *Magnetic Resonance in Medicine*.

